# Phosphorylation of SARS-CoV-2 Orf9b Regulates Its Targeting to Two Binding Sites in TOM70 and Recruitment of Hsp90

**DOI:** 10.3390/ijms22179233

**Published:** 2021-08-26

**Authors:** Lukas Brandherm, Antonio Mario Kobaš, Mara Klöhn, Yannick Brüggemann, Stephanie Pfaender, Joachim Rassow, Sebastian Kreimendahl

**Affiliations:** 1 Institute for Biochemistry and Pathobiochemistry, Ruhr-University Bochum, 44801 Bochum, Germany; lukas.brandherm@rub.de (L.B.); antonio.kobas@rub.de (A.M.K.); sebastian.kreimendahl@rub.de (S.K.); 2 Department of Molecular & Medical Virology, Ruhr-University Bochum, 44801 Bochum, Germany; Mara.Kloehn@rub.de (M.K.); Yannick.brueggemann@rub.de (Y.B.); stephanie.pfaender@rub.de (S.P.)

**Keywords:** SARS-CoV-2, COVID19, B.1.1.7, variant of concern, Orf9b, TOM70, mitochondria, Hsp90, interferon

## Abstract

SARS-CoV-2 (severe acute respiratory syndrome coronavirus 2) is the causative agent of the COVID19 pandemic. The SARS-CoV-2 genome encodes for a small accessory protein termed Orf9b, which targets the mitochondrial outer membrane protein TOM70 in infected cells. TOM70 is involved in a signaling cascade that ultimately leads to the induction of type I interferons (IFN-I). This cascade depends on the recruitment of Hsp90-bound proteins to the N-terminal domain of TOM70. Binding of Orf9b to TOM70 decreases the expression of IFN-I; however, the underlying mechanism remains elusive. We show that the binding of Orf9b to TOM70 inhibits the recruitment of Hsp90 and chaperone-associated proteins. We characterized the binding site of Orf9b within the C-terminal domain of TOM70 and found that a serine in position 53 of Orf9b and a glutamate in position 477 of TOM70 are crucial for the association of both proteins. A phosphomimetic variant Orf9b_S53E_ showed drastically reduced binding to TOM70 and did not inhibit Hsp90 recruitment, suggesting that Orf9b–TOM70 complex formation is regulated by phosphorylation. Eventually, we identified the N-terminal TPR domain of TOM70 as a second binding site for Orf9b, which indicates a so far unobserved contribution of chaperones in the mitochondrial targeting of the viral protein.

## 1. Introduction

Since the beginning of the COVID19 pandemic, its causative agent, SARS-CoV-2 (severe acute respiratory syndrome coronavirus 2), has evolved into several lineages. The SARS-CoV-2 variant of concern (VoC) Alpha (B.1.1.7, VOC 202012/01) emerged in England in September 2020 and, within a few months, became the worldwide dominating lineage [1]. A hallmark of VoC Alpha is a higher transmissibility compared to preexisting virus variants, and recent studies indicate that it also poses a higher risk of hospitalization and ICU admission [2,3,4,5,6]. VoC Alpha is characterized by several genomic mutations, one of which is found within its N gene and seems to increase the expression of a sub-genomic open reading frame (ORF) encoding a small accessory protein termed Orf9b [7,8]. Recent unpublished data suggest that a sub-genomic RNA (sgRNA) encoding for Orf9b is expressed up to 16-fold in samples of patients infected with VoC Alpha, hinting at an important role of Orf9b for the pathogenicity of this variant [9].

Orf9b interacts with the mitochondria of infected cells by binding to TOM70, a versatile adaptor protein of the mitochondrial outer membrane involved in many cellular processes, including protein import, mediation of membrane contacts, and the innate immune response to viral infections [10,11]. Expression of Orf9b leads to a decreased induction of type I interferons (IFN-I) in infected cells by interfering with a signaling cascade involving the proteins RIG-I, MAVS, TOM70, TBK1, and IRF3 [12,13,14]. Upon viral RNA recognition, RIG-I binds to MAVS, which, in turn, interacts with TOM70 to allow association with Hsp90-bound TBK1 and IRF3. It has been speculated that inhibition of Hsp90 binding by Orf9b could be a possible reason for decreased IFN-I expression [13]. A study using isothermal titration calorimetry (ITC) indicated that the binding of an Orf9b-derived peptide inhibits the binding of an Hsp90-derived peptide to the N-terminal domain of TOM70, suggesting an allosteric inhibition [15]. However, the influence of Orf9b binding on the recognition of native Hsp90 was not investigated.

A high-resolution Cryo-EM structure of a core fragment of Orf9b in complex with human TOM70 depicts the central part of Orf9b (amino acids 39–76) buried within the C-terminal domain of TOM70 [16]. Additionally, a crystal structure of a similar fragment of Orf9b (amino acids 43–78) in complex with human TOM70 was published recently and confirmed the localization of the core part of Orf9b within TOM70 [15]. Within the interaction interface of the Orf9b–TOM70 complex, both structures show a prominent amino acid of Orf9b, a serine at position 53 (S53), to form a hydrogen bond with a glutamate at position 477 (E477) of TOM70 in an overall hydrophobic region. Interestingly, S53 was identified as an infection-driven phosphorylation site, suggesting a possible mechanism of regulation of the Orf9b–TOM70 interaction [17].

In this study, we characterized the binding site of Orf9b within the C-terminal domain of TOM70 and found that the interaction of Orf9b S53 with TOM70 E477 is crucial for complex formation. A phosphomimetic variant, Orf9b_S53E_, showed dramatically decreased incorporation into the C-terminal domain of TOM70, suggesting that the binding of Orf9b to the receptor can be regulated by the phosphorylation of Orf9b S53. We found that the binding of Orf9b inhibits the association of TOM70 with the cytosolic chaperone Hsp90, explaining the observed decrease in the IFN-I response in Orf9b-expressing cells. Eventually, we identified a second binding site of Orf9b within the N-terminal TPR domain of TOM70, defined by the arginine in position 192 (R192), which promotes the binding of Orf9b to the central part of TOM70.

## 2. Results

### 2.1. A Phosphomimetic Amino Acid Exchange in Orf9b Prevents Its Interaction with TOM70

**Figure 1:**
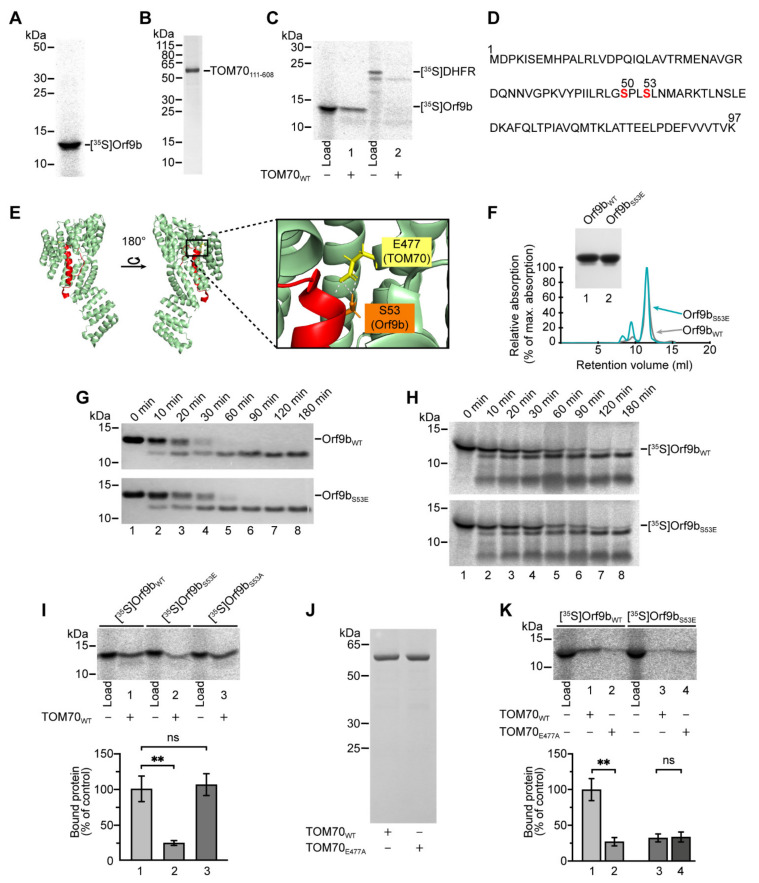
Characterization of Orf9b binding to the C-terminal domain of TOM70. (**A**) Radiolabeled SARS-CoV-2 [^35^S]Orf9b_WT_ was synthesized in rabbit reticulocyte lysate and was analyzed by SDS-PAGE. (**B**) Coomassie staining of TOM70_111–608_ purified using Ni-NTA agarose beads. (**C**) In vitro binding of [^35^S]Orf9b_WT_ or [^35^S]DHFR (mouse dihydrofolate reductase) to 250 pmol Ni-NTA bound TOM70_111–608_. Signals obtained after SDS-PAGE and autoradiographic detection were quantified using 2% of the total added lysate as internal control (“Load”). (**D**) Primary structure of SARS-CoV-2 Orf9b highlighting the serine residues at position 50 and 53 of Orf9b. (**E**) Structure of the Orf9b–TOM70 complex highlighting the interaction of serine 53 of Orf9b with glutamate 477 of TOM70. Structural data were obtained from PDB file 7KDT [16]. (**F**) Size-exclusion chromatography of Orf9b_WT_ and Orf9b_S53E_ using a Superdex S75 increase 10/300 GL column depicting normalized UV absorption. DHFR-fused Orf9b variants were expressed in *E. coli* and purified from cell lysates by IMAC, IEC, removal of the DHFR tag by TEV digestion with subsequent affinity purification and SEC (Insert). (**G**) Trypsin treatment of purified Orf9b_WT_ and Orf9b_S53E_ yielded identical degradation products. For trypsin digestion, 5.5 µg/mL Orf9b_WT_ or Orf9b_S53E_ were incubated with 5 µg/mL trypsin on ice for the indicated times, and the reaction was stopped by incubation at 95 °C for 5 min. Samples were subsequently analyzed by SDS-PAGE and visualized by Coomassie staining. (**H**) Radiolabeled [^35^S]Orf9b_WT_ and [^35^S]Orf9b_S53E_ synthesized in reticulocyte lysate show similar trypsin degradation products. Trypsin digestion was performed as described in G, using 20% (*v/v*) reticulocyte lysate containing [^35^S]-labeled protein instead of purified proteins. (**I**) In vitro binding of radiolabeled Orf9b variants to Ni-NTA bound TOM70_111-608_. The experiment was carried out as described in C, and binding of [^35^S]Orf9b_WT_ was set as control. Signals obtained after SDS-PAGE and autoradiographic detection were quantified using 2% of the total added lysate as internal control (“Load”). ** indicate *p*-values < 0.01, “ns” indicates no significance, error bars show SEM, *n* = 4. (**J**) Coomassie staining of TOM70_WT_ and TOM70_E477A_ purified using Ni-NTA agarose beads. (**K**) In vitro binding of radiolabeled [^35^S]Orf9b_WT_ and [^35^S]Orf9b_S53E_ to 250 pmol of either TOM70_WT_ or TOM70_E477A_ was carried out as described in (C). Orf9b_WT_ binding to TOM70_WT_ was set to 100% (control). ** indicate *p*-values < 0.01, “ns” indicates no significance, error bars show SEM, *n* = 4.

In previous studies, the complex formation of Orf9b and TOM70 was mainly characterized using purified proteins or synthetic peptides [15,16]. To investigate the interaction of both proteins under the conditions of a mammalian cytosol, we translated Orf9b in rabbit reticulocyte lysate (RL) in the presence of [^35^S]-methionine (Figure 1A). This system includes all cytosolic factors and molecular chaperones that may participate in the targeting and binding of proteins to the mitochondrial surface [18,19,20,21,22]. We then used samples of the reticulocyte lysate containing radiolabeled Orf9b for incubation with the cytosolic domain of human TOM70 (amino acids 111–608). For this purpose, TOM70 was bound to Ni-NTA agarose beads, following a basic protocol published by Brix et al. [23] (Figure 1B). The presence of equal amounts of TOM70 in the individual samples was confirmed by elution of the Ni-NTA-bound protein and Western blot analysis using an antibody against the N-terminal His tag of TOM70 (Figure S1). Using this system, we found that radiolabeled Orf9b associated with TOM70 (Figure 1C, lane 1). Under the same conditions, radiolabeled mouse dihydrofolate reductase (DHFR), a cytosolic protein of 21.6 kDa, often used as an established model protein [24,25,26], showed no binding to the receptor (Figure 1C, lane 2).

A recent study identified two phosphorylation sites on Orf9b: a serine at position 50 (S50) and a serine at position 53 (S53) (Figure 1D) [17]. Orf9b S53 forms a prominent hydrogen bond with a conserved glutamate at position 477 in the C-terminal domain of TOM70 (E477) within the overall hydrophobic interaction interface of both proteins (Figure 1E) [15,16]. We hypothesized that the phosphorylation of Orf9b S53 would destabilize the interaction with TOM70 E477, which, in turn, could weaken the association of both proteins. To test for the consequences of the phosphorylation of S53, we replaced serine 53 with a phosphomimetic glutamate residue, leading to the variant Orf9b_S53E_. Since Orf9b forms homodimers in the absence of TOM70 [15,16,27], we tested for a possible influence of the mutation of S53 on protein multimerization by purifying both variants Orf9b_WT_ and Orf9b_S53E_ from *E. coli* (Figure 1F, insert). The comparison with a standard curve of proteins of known molecular mass indicated that during size exclusion chromatography (SEC), both Orf9b variants were eluted as dimers of a molecular mass of approximately 28.7 kDa (Figure 1F). To exclude that the introduction of the phosphomimetic residue leads to structural changes within Orf9b, we incubated the purified variants in the presence of trypsin and subsequently compared the obtained digestion pattern of degradation products by SDS-PAGE. We observed a time-dependent appearance of a trypsin-resistant fragment of Orf9b for both variants, suggesting that the tertiary structures of the proteins were similar (Figure 1G). To exclude a negative effect of the S53E mutation on a possible association of Orf9b with cytosolic factors, we synthesized the two variants, Orf9b_WT_ and Orf9b_S53E_, in the in vitro translation system and repeated the experiment. We found a similar appearance of the trypsin-dependent Orf9b fragment for both variants, suggesting that interaction with cytosolic factors is not mitigated for Orf9b_S53E_ (Figure 1H).

We then used the binding assay to compare the interactions of radiolabeled Orf9b_WT_ and Orf9b_S53E_ with Ni-NTA-bound TOM70 (Figure 1I). We found that the binding of Orf9b_S53E_ to isolated TOM70 was reduced by 76% compared to the wild-type protein (Figure 1I, lane 1 vs. lane 2). Conversely, the binding of a variant in which S53 was replaced by an alanine, Orf9b_S53A_, showed no reduction in TOM70 binding, demonstrating the specificity of this effect for the phosphomimetic variant (Figure 1I, lane 1 vs. lane 3).

To further characterize the effects of the different residues in position 53, we fused each variant of Orf9b to DHFR (to facilitate the resolution in SDS-PAGE), synthesized the hybrid proteins in reticulocyte lysate, and tested for the accessibility of trypsin cleavage sites in the presence or absence of purified soluble TOM70. In the absence of TOM70, trypsin treatment of radiolabeled Orf9b_WT_-DHFR in the reticulocyte lysate yielded a specific fragment corresponding to an N-terminally truncated product (Figure S2, fragment f1, lanes 2–4). In the presence of TOM70, we again observed an N-terminally shortened degradation product, albeit of higher molecular mass. This indicates that Orf9b_WT_-DHFR is efficiently bound to the C-terminal domain of TOM70, with most parts of the construct being shielded from the protease (Figure S2, fragment f2, lanes 6–8). The same pattern was obtained with Orf9b_S53A_-DHFR, indicating that the access of this construct to the binding site in the C-terminal domain of TOM70 was retained (Figure S2, fragment f2, lanes 6–8). In contrast, Orf9b_S53E_-DHFR was degraded to the same fragments, both in the presence and absence of TOM70, confirming that this variant is not incorporated into the receptor (Figure S2, fragment f1, lanes 6−7).

To further characterize the interaction between Orf9b S53 and TOM70 E477, we tested the binding of radiolabeled Orf9b_WT_ and Orf9b_S53E_ to isolated wild-type TOM70 (TOM70_WT_) and to a variant, in which the glutamate at position 477 was exchanged for an alanine (TOM70_E477A_; Figure 1J). The binding of in vitro-synthesized Orf9b_WT_ to TOM70_E477A_ was reduced by 73% in comparison to wild-type TOM70, thus to a similar extent as observed for the binding of Orf9b_S53E_ to TOM70_WT_ (Figure 1K, lane 2 vs. lane 3). This suggests that the interaction of the serine S53 in Orf9b with the glutamate E477 in TOM70 is crucial for the formation of the Orf9b–TOM70 complex since a mutation of either amino acid residue drastically reduces the association of the two proteins. Interestingly, the incubation of phosphomimetic Orf9b_S53E_ with isolated TOM70_E477A_ did not lead to a reduction in comparison to TOM70_WT_, indicating that the reduced association of Orf9b_S53E_ with TOM70_WT_ is not simply a result of the repulsion of two negative charges (Figure 1K, lane 3 vs. lane 4).

**Figure 2:**
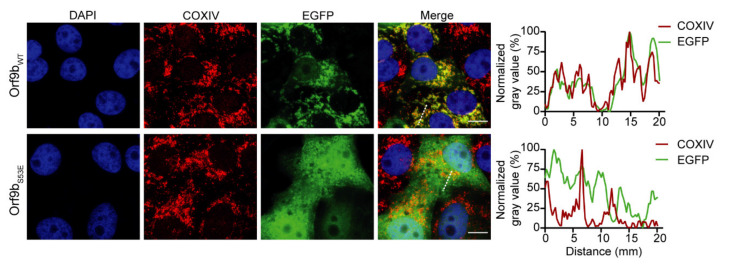
Subcellular localization of Orf9b_WT_ and Orf9b_S53E_. Representative immunofluorescence images of Vero E6 cells expressing Orf9b_WT_-EGFP or Orf9b_S53E_-EGFP 24 h post-transfection (left panels). Gray value profiles of EGFP (green curves) and COXIV (red curves) (right) were measured with Image J (Fiji) software and normalized to the smallest and largest values in each data set. Gray value profiles were plotted along the white dotted lines. Cell nuclei are shown in blue (DAPI) and mitochondria in red. Scale bars, 10 µm.

To test the relevance of our findings in mammalian cell culture, we expressed the variants Orf9b_WT_ and Orf9b_S53E_ C-terminally fused to EGFP in Vero E6 cells, and determined their cellular distribution by fluorescence microscopy (Figure 2). COXIV, a subunit of the cytochrome c oxidase (complex IV of the respiratory chain), was immunostained for the visualization of mitochondria. While Orf9b_WT_ showed a strong co-localization with mitochondria, the phosphomimetic variant Orf9b_S53E_ displayed a diffuse pattern, indicating a cytosolic location for the majority of molecules (Figure 2, profiles). Notably, we observed some co-localization of Orf9b_S53E_-EGFP with the DAPI-stained nuclei of several cells, which is likely a result of the lack of specific targeting of this construct.

The binding of Orf9b to TOM70 is obviously highly dependent on the interaction of serine 53 of Orf9b with glutamate 477 of TOM70. Our data further suggest that the association of both proteins is inhibited upon the phosphorylation of S53 of Orf9b.

### 2.2. Binding of Orf9b Inhibits the Interaction of TOM70 with Cytosolic Hsp90

**Figure 3:**
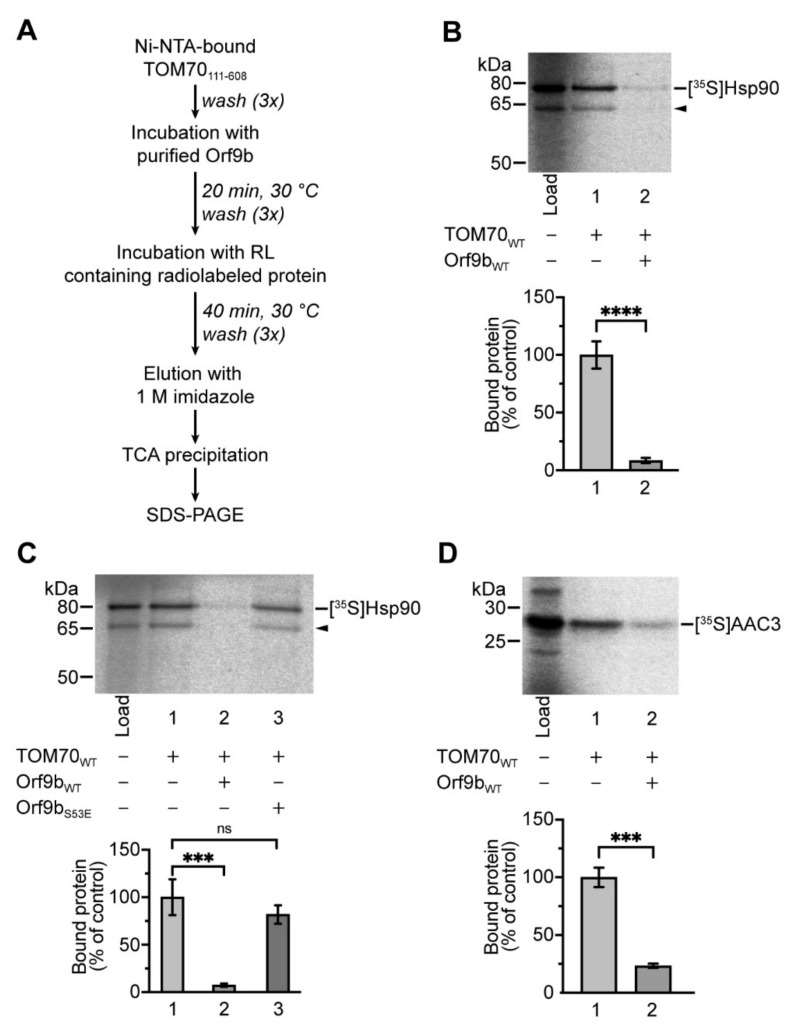
Orf9b inhibits the binding of native Hsp90 to TOM70 in a phosphorylation-dependent manner. (**A**) Workflow of the binding assay used to investigate the effects of pre-incubation with Orf9b variants on the binding of native TOM70_111–608_ interactors. (**B**) Influence of Orf9b binding to TOM70 on the recognition of Hsp90. Ni-NTA-bound TOM70_111–608_ (50 pmol) was pretreated either with a 10-fold molar excess of isolated Orf9b_WT_ or with buffer for 20 min at 30 °C and washed three times before addition of reticulocyte lysate (RL) containing in vitro-synthesized human [^35^S]Hsp90. Samples were incubated for 40 min at 30 °C and subjected to SDS-PAGE. The arrow indicates a second translation product of Hsp90. Binding of Hsp90 to TOM70_111–608_ without the addition of Orf9b was set to 100% (control). Signals obtained after SDS-PAGE and autoradiographic detection were quantified using 5% of the total added lysate as internal control (“Load”). **** indicate *p*-values < 0.0001, error bars show SEM, *n* = 6. (**C**) Binding of [^35^S]Hsp90 to Ni-NTA-bound TOM70_111–608_, pretreated with either buffer, isolated Orf9b_WT_, or isolated Orf9b_S53E_ was performed as described in (**B**). Signals obtained after SDS-PAGE and autoradiographic detection were quantified using 5% of the total added lysate as internal control (“Load”). Binding of [^35^S]Hsp90 without Orf9b pre-incubation was set to 100% (control). *** indicate *p*-values < 0.001, “ns” indicates no significance, error bars show SEM, *n* = 4. (**D**) In vitro binding of [^35^S]AAC3 to Ni-NTA-bound TOM70_111–608_ pre-incubated with or without Orf9b_WT_. The experiment was carried out as described in (**B**). Signals obtained after SDS-PAGE and autoradiographic detection were quantified using 10% of the total added lysate as internal control (“Load”). Binding of [^35^S]AAC3 without Orf9b_WT_ pre-incubation was set to 100% (control). *** indicate *p*-values < 0.001, error bars show SEM, *n* = 3.

TOM70 is part of a signaling cascade that ultimately leads to the expression of IFN-I in response to RNA virus infections [11]. A crucial part of this cascade is the interaction of MAVS with TOM70 on the mitochondrial surface, which enables subsequent binding of Hsp90-bound TBK1 and IRF3 [12]. A previous study has shown that SARS-CoV-2 Orf9b targets TOM70 in mammalian cells and diminishes the expression of IFN-β, leading the authors to speculate that the binding of Orf9b to TOM70 might interfere with the recognition of Hsp90 [13]. A recent study determined the binding affinities of synthetic peptides to TOM70 by isothermal titration calorimetry (ITC) and found that an association of a short peptide containing the C-terminal EEVD motif of Hsp90 with the N-terminus of TOM70 was greatly decreased if the receptor was in complex with co-purified Orf9b [15]. Mammalian cytosol contains two isoforms of Hsp90, an inducible isoform, Hsp90-α, and the constitutively expressed isoform Hsp90-β [28]. To investigate if Orf9b binding to TOM70 affects its association with native full-length human Hsp90-β, we again used the procedure described by Brix et al. [23]. To this end, we pre-incubated isolated TOM70 with either purified Orf9b_WT_ or Orf9b_S53E_ and subsequently added reticulocyte lysate containing radiolabeled human Hsp90 (Figure 3A). The lysate contained a distinct second translation product of lower molecular mass, most likely caused by a second in-frame start codon within the open reading frame (Figure 3B, black arrow) [29]. The ability of this second product to associate with TOM70 is in agreement with the notion that complex formation is independent of the N-terminal part of Hsp90 and primarily mediated by its C-terminal EEVD motif, which binds to a tetratricopeptide repeat (TPR) domain within the N-terminus of TOM70 [30,31]. We found that the pre-incubation with Orf9b_WT_ inhibited subsequent binding of Hsp90 almost completely (Figure 3B). No effect was observed when TOM70 was pre-incubated with the phosphomimetic variant Orf9b_S53E_, demonstrating that the interaction of S53 of Orf9b with E477 of TOM70 is essential not only for stable insertion into the Orf9b binding site but also for the inhibitory effect on the recruitment of Hsp90 (Figure 3C).

TOM70 is known to act as a receptor for newly synthesized mitochondrial ADP/ATP carriers and related proteins of the same protein family [11]. In the cytosol, these preproteins are bound by chaperone proteins and, thereby, retained in a soluble state [18,19]. We recently found that in the mitochondrial import of carrier proteins, TOM70 functions essentially as a co-chaperone [24]. To test for possible effects of Orf9b on this system, we pre-incubated TOM70 with Orf9b_WT_ and subsequently added reticulocyte lysate containing radiolabeled human ADP/ATP carrier 3 (AAC3), a model protein for chaperone-mediated interaction with TOM70. We found that Orf9b binding to TOM70 drastically reduced the binding of AAC3 to TOM70 by 77%, indicating a general inhibition of chaperone-bound TOM70 substrates (Figure 3D).

### 2.3. The Chaperone Binding Site of TOM70 Promotes Association with Orf9b

TOM70 contains a chaperone-binding site within its N-terminal part, including an important arginine residue in position 192 [31,32]. Considering the involvement of chaperones in the mitochondrial targeting of newly synthesized proteins, we reasoned that this chaperone-binding site may participate in the recognition of Orf9b. In a previous study, co-immunoprecipitation experiments were carried out with a TOM70 variant lacking the corresponding N-terminal TPR domain [13]. The results showed that Orf9b was still able to bind to this construct, suggesting a mechanism of direct access of Orf9b to the binding site in the C-terminal part of TOM70. However, using the cytosolic domain of TOM70 (residues 111–608), we found that upon disruption of this site (defined by residue E477) or by exchange of the corresponding residue within Orf9b (serine 53), a reduced but still significant association of both proteins was retained (Figure 1K).

To test if Orf9b may be a candidate for an attachment to the TOM70 chaperone binding site, we investigated the state of Orf9b in reticulocyte lysate. It is well established that mitochondrial precursor proteins translated in rabbit reticulocyte lysate form high molecular weight complexes of approximately 200–250 kDa that contain cellular chaperones [21,22]. We analyzed samples of reticulocyte lysate containing radiolabeled Orf9b by blue native polyacrylamide gel electrophoresis (BN-PAGE). Remarkably, two distinct states of the translation product were separated, with most of the Orf9b corresponding to a complex of approximately 220–240 kDa (Figure S3, labeled **) and smaller amounts being resolved at a lower molecular weight. Orf9b_S53E_ showed slightly higher mobility in native gels compared to Orf9b_WT_, presumably because of the additional negative charge of Orf9b_S53E_ (data not shown). A signal within the range of 200–250 kDa was not detected with the cytosolic protein DHFR, a small hydrophilic protein that is soluble independently of a permanent association with chaperone proteins (Figure S3, labeled **).

**Figure 4:**
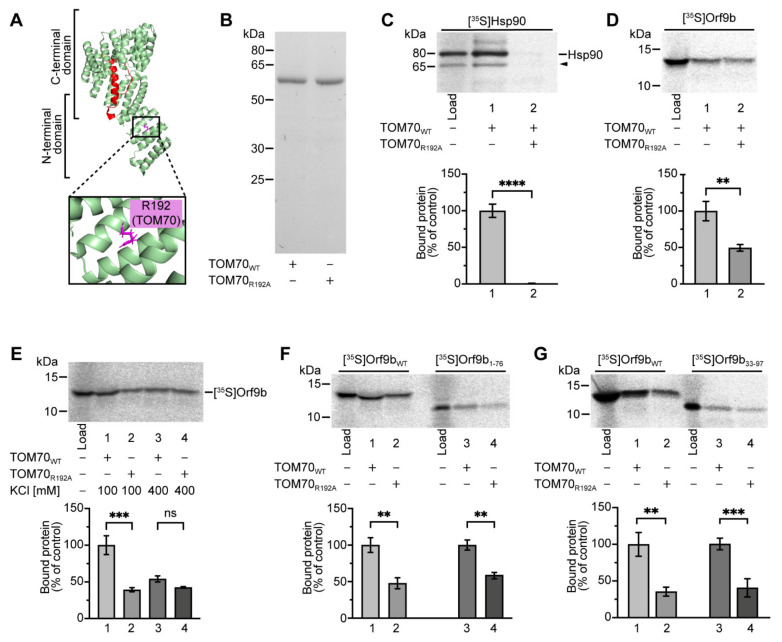
Orf9b has a second binding site in the N-terminal TPR domain of TOM70. (**A**) Overview of the N-terminal chaperone binding site of TOM70 defined by arginine R192. Structural data were obtained from PDB file 7KDT [16]. (**B**) Coomassie staining of TOM70_WT_ and TOM70_R192A_ purified using Ni-NTA agarose beads. (**C**) In vitro binding of [^35^S]Hsp90 to 50 pmol Ni-NTA-bound TOM70_111–608_ WT (TOM70_WT_) or TOM70_111–608_ R192A (TOM70_R192A_). Binding of [^35^S]Hsp90 to TOM70_WT_ was set to 100% (control). **** indicate *p*-values < 0.0001, error bars show SEM, *n* = 7. (**D**) Binding of [^35^S]Orf9b to 250 pmol Ni-NTA-bound TOM70_WT_ or TOM70_R192A_. Binding of [^35^S]Orf9b to TOM70_WT_ was set to 100% (control). ** indicate *p*-value < 0.01, error bars show SEM, *n* = 5. (**E**) Binding of [^35^S]Orf9b_WT_ to 250 pmol Ni-NTA-bound TOM70_WT_ or TOM70_R192A_ under high (400 mM KCl) or normal (100 mM KCl) salt conditions. Binding of [^35^S]Orf9b_WT_ to TOM70_WT_ in 100 mM KCl was set to 100% (control). *** indicate *p*-values < 0.001, “ns” indicates no significance, error bars show SEM, *n* = 3. (**F**) In vitro binding of [^35^S]Orf9b_WT_ and [^35^S]Orf9b_1–76_ to 250 pmol Ni-NTA-bound TOM70_WT_ or TOM70_R192A_. Binding of Orf9b_WT_ or Orf9b_1–76_ to TOM70_WT_ was set to 100% (control). ** indicate *p*-values < 0.01, error bars show SEM, *n* = 5. (**G**) In vitro binding of [^35^S]Orf9b_WT_ and [^35^S]Orf9b_33–97_ to TOM70_WT_ and TOM70_R192A_. Binding of Or9fb_WT_ or Orf9b_33–97_ to TOM70_WT_ was set to 100% (control). ** indicate *p*-values < 0.01; *** indicate *p*-values < 0.001, error bars show SEM, *n* = 9.

To investigate the possible participation of cytosolic chaperones in the formation of the Orf9b–TOM70 complex, we purified an established model protein, TOM70_R192A_, in which an arginine residue at position 192 within its N-terminal TPR domain is exchanged for an alanine to prevent the interaction of TOM70 with the C-terminal EEVD motif of Hsp70/Hsp90 chaperones (Figure 4A) [31]. We purified the variants TOM70_WT_ and TOM70_R192A_ using Ni-NTA beads (Figure 4B) and confirmed that the mutation R192A within TOM70 prevents the binding of Hsp90 in our assay (Figure 4C). In parallel, we also observed a reduction of binding, between 50% (Figure 4D) and 65% (Figure 4G), of Orf9b to TOM70_R192A_ in comparison to TOM70_WT_. The results of these assays show that the chaperone binding site within the N-terminal domain of TOM70 is not essential for the recruitment of Orf9b; however, the binding of Orf9b is substantially facilitated.

The binding of chaperones to the N-terminal domain of TOM70 is mediated by hydrophilic residues [31,32]. To verify the role of the chaperone binding site, we tested for the association of Orf9b with TOM70 under different salt concentrations. We found that a high salt concentration (400 mM KCl) reduced the binding of Orf9b by about 55% compared to normal salt levels (100 mM KCl), presumably by weakening the hydrophilic interactions in the N-terminus of TOM70 (Figure 4E, lane 1 vs. lane 2). In contrast, this effect was not observed using the variant TOM70_R192A_ (lacking an intact chaperone binding site). Using this variant, the binding of Orf9b was not significantly reduced under high salt conditions (Figure 4E, lane 3 vs. lane 4). Additionally, we found that the core fragment of Orf9b (amino acids 33–76) was sufficient for chaperone-mediated binding as Orf9b variants lacking either the C-terminus (amino acids 77–97, Figure 4F) or the N-terminus (amino acids 1–32, Figure 4G) were still affected by the mutation of arginine 192 within the chaperone binding site of TOM70.

Our results indicate that interaction of Orf9b with TOM70 is mediated by two distinct binding sites: (i) a site within the C-terminal domain of TOM70 (defined by Orf9b S53 and TOM70 E477), and (ii) a second binding site within the N-terminal TPR domain of TOM70 (defined by TOM70 R192 and the EEVD motif of Orf9b-associated chaperones). While the C-terminal binding site is crucial for the interaction of Orf9b with TOM70, the N-terminal binding site appears to promote the binding of chaperone-associated Orf9b.

## 3. Discussion

### 3.1. The Mechanisms of Orf9b–TOM70 Interaction

Orf9b is a SARS-CoV-2-encoded accessory protein that can bind to a defined site within the bundle of helices that form the C-terminal part of the mitochondrial outer membrane protein TOM70 [15,16]. In this study, we investigated the process of complex formation and its functional implications.

We synthesized Orf9b in reticulocyte lysate and, thus, under conditions similar to the cytosol of a mammalian cell. Using BN-PAGE, we analyzed samples of the reticulocyte lysate and found that most of the Orf9b was a component of a high molecular weight complex of 220–240 kDa, corresponding to the size of newly synthesized chaperone-bound preproteins that are retained in a soluble state for subsequent import into mitochondria [21,22]. In mitochondrial protein import, TOM70 acts as a co-chaperone by binding chaperone-associated preproteins to facilitate the recognition of their targeting signals by the translocase of the outer membrane (TOM) complex [24]. In human TOM70, the interaction site for the recruitment of the chaperone-associated preproteins is defined by an arginine residue in position 192 within the N-terminal part of TOM70 [31]. In our experiments, we found that an exchange of this residue for alanine (R192A) reduced the efficiency of Orf9b binding to TOM70 by about 50%. Remarkably, this effect corresponds to similar values with regard to the contribution of TOM70 in the import of proteins into the inner mitochondrial compartments [24,33,34]. For efficient import of nuclear-encoded proteins into mitochondria, TOM70 is not essential; however, it substantially facilitates the targeting of its substrate proteins. Subsequent translocation across the outer membrane is mediated by the pore-forming protein TOM40 [11,18,19,35,36,37]. However, after release from the chaperone, Orf9b leaves the import pathway and is transferred to the binding site in the C-terminal part of TOM70. This final step can be inhibited by an exchange of the central glutamate E477 within this binding site. Under these conditions, the association of Orf9b with the modified TOM70 is drastically reduced by about 75%, but a substantial residual binding is still observed.

In summary, Orf9b initially follows a pattern that closely resembles the pathway of endogenous mitochondrial proteins that are imported from the cytosol. Orf9b diverges from this scheme after its initial interaction with the chaperone binding site at the arginine R192 of TOM70 to enter the binding cleft within its C-terminal domain. Orf9b is thus a protein with two binding sites within the same receptor protein.

The genome of the yeast *Saccharomyces cerevisiae* encodes two homologs of human TOM70 [11]. A comparison of the crystal structures of yeast Tom70 and its paralog Tom71 indicates that both receptors can exist in at least two distinct conformational states, an elongated “open” state and a “closed” state [11,38,39,40]. In yeast, the binding of the C-terminal EEVD motif of Hsp70 was suggested to induce the transition of Tom71 to its “open” state [39]. It is tempting to speculate that the binding of Orf9b to the N-terminal TPR domain of human TOM70, defined by the arginine at position 192, may trigger a switch to an “open” conformation of the receptor. This would allow for subsequent interaction of Orf9b with its binding site within the C-terminal domain of TOM70, defined by the glutamate E477. It is conceivable that Orf9b binding locks the receptor in its “open” state to prevent interaction with other Hsp90-bound proteins. In fact, we found that the binding of AAC3, a nuclear-encoded mitochondrial preprotein, to human TOM70 was drastically reduced under these conditions.

In parallel experiments, we also investigated the binding of isolated Orf9b to purified TOM70. Previous studies had suggested that Orf9b purified from *E. coli* is not able to associate with TOM70 in vitro [15,16]. Surprisingly, we found that the binding of isolated Orf9b to TOM70 is indeed possible when added in molar excess (1:10). Obviously, in the absence of chaperone proteins or additional factors, only a small fraction of the readily available Orf9b has the ability to bind to TOM70 in vitro. Notably, while purified dimeric Orf9b mainly forms β-strands, monomeric Orf9b displays an overall α-helical structure when in complex with TOM70 [15,16,27]. The ability of Orf9b to switch folds is also indicated by structure- and sequence-based predictions [41]. It is, therefore, tempting to speculate that a portion of Orf9b can undergo dynamic shifts between its dimeric state and its monomeric state.

### 3.2. Implications of Orf9b Binding to TOM70 for Health and Disease

SARS-CoV-2 VoC Alpha, also known as lineage B.1.1.7, is characterized by a higher transmissibility than preexisting virus variants [2,3,4]. Interestingly, recent data suggest a higher expression of Orf9b in cells of patients infected with the Alpha lineage and that this variant could lead to a higher number of hospitalizations and severe disease [5,6,9]. While it seems unlikely that an increased Orf9b expression plays a role in person-to-person transmission, it is conceivable that it represents a way to inhibit the innate immune response, which could provide a significant advantage over other virus variants. Previous studies have shown that expression of Orf9b in human cells leads to a reduced expression of IFN-I by the inhibition of a signaling cascade involving the proteins RIG-I, MAVS, TOM70, and Hsp90-bound TBK1 and IRF3 [13,14]. A compromised innate immune response has been suggested to be an important driver of COVID19, making the Orf9b–TOM70 interaction an attractive target for the development of therapeutic strategies [42]. In this regard, the mechanism of interference with the IFN-I inducing signaling cascade is of the utmost interest. Although it was concluded from experiments with short peptides derived from Hsp90 and Orf9b that the inhibition of Hsp90 binding to TOM70 could be a possible reason for the reduced IFN-I expression, the interplay of the three native proteins has not yet been investigated [15].

In this study, we found that the binding of Orf9b to TOM70 prevents the recognition of native Hsp90 and chaperone-associated TOM70 substrates. Due to technical limitations, we did not investigate the binding of a complex consisting of Hsp90/TBK1/IRF3; however, our data strongly suggest that inhibition of the interaction of Hsp90 with TOM70 represents the reason for the decreased IFN-I response in infected cells. Additionally, we could show that the substitution of the serine at position 53 by a phosphomimetic glutamate residue in Orf9b prevented its interaction with the C-terminal domain of TOM70 in vitro and in intact mammalian cells. During the preparation of this manuscript, a preprint that was published confirmed our finding that Orf9b binding is dependent on the state of its serine in position 53 [43]. In addition, the authors observed the suppression of host kinase activity in the early stages of infection, which likely represents a way of innate immune evasion for the virus.

Our data indicate that not only Hsp90 itself but also Hsp90-bound proteins are affected by the inhibitory effect of Orf9b, as suggested by the drastically reduced binding of in vitro-synthesized AAC3 to TOM70. The formation of the Orf9b–TOM70 complex may have additional implications for cellular processes beyond innate immunity, for example, in the recruitment of chaperone-bound preproteins [11,24,44]. In this context, it is interesting that peripheral blood mononuclear cells (PBMCs) from COVID19 patients seem to show metabolic alterations and reduced respiration that are compensated by a switch to glycolysis [45]. Therefore, the interaction of mitochondria with SARS-CoV-2-encoded proteins represents a promising avenue for further research with tremendous therapeutic potential.

The interaction of Orf9b with TOM70 will be an attractive target for the development of novel therapeutic strategies; however, it seems important to take into account the participation of two different binding sites in the formation of the Orf9b–TOM70 complex.

## 4. Materials and Methods

### 4.1. Plasmids

The plasmids used in this study are listed in Table 1. For the construction of the plasmids, DNA fragments were amplified by a standard PCR protocol or a protocol modified for FastCloning, as described by Li et al. (2011) [46], using Phusion DNA Polymerase (Thermo Scientific, Waltham, MA, USA). After restriction enzyme digestion, inserts were either ligated into vectors using T4 DNA Ligase (Thermo Scientific, Waltham, MA, USA) or mixed with DpnI-digested vector backbone prior to transformation in *E. coli* TOP10 (Thermo Scientific, Waltham, MA, USA).

### 4.2. Protein Purification

Orf9b was isolated from *E. coli* using the protocol published by Gordon et al. (2020) with minor modifications [16]. *E. coli* BL21 (DE3) cells were transformed with pProEx plasmids encoding for His-DHFR-TEV-Orf9b (His-DHFR fused to Orf9b via a TEV protease cleavage site) and tested for protein expression. Expression of His-DHFR-TEV-Orf9b was induced by the addition of 1 mM IPTG and carried out for 6 h at 37 °C. Cells were harvested at 6000× *g* for 5 min, frozen in appropriate aliquots, and stored at −70 °C until further use. After thawing, cells were resuspended in 10 mL/g lysis buffer (50 mM Tris/HCl; 500 mM NaCl; 10% (*v/v*) glycerol; 2 mM MgCl_2_; 2 mM PMSF; pH = 8.0) supplemented with 4.5 U/mL DNase I and protease inhibitors and incubated for 30 min at 4 °C. Complete lysis was obtained after four passages through an Emulsiflex C5 homogenizer (Avestin, Ottawa, ON, Canada). The lysate was cleared from cell debris by centrifugation at 47,500× *g* for 30 min. The supernatant was supplemented with 20 mM imidazole and loaded on two consecutive 1 mL Ni-NTA HisTrap High Performance columns (Sigma-Aldrich, St. Louis, MO, USA) equilibrated with lysis buffer using an ÄKTA start protein purification system (Cytiva, Marlborough, MA, USA). Columns were washed with 10 column volumes (cv) wash buffer (30 mM Tris/HCl; 150 mM KCl; 500 mM NaCl; 10% (*v/v*) glycerol; 0.2% Tween20; 2 mM ATP; 4 mM MgCl_2_; 40 mM imidazole; 1 mM DTT) followed by 5 cv buffer A (25 mM Tris/HCl, 50 mM KCl, 5% (*v/v*) glycerol; 1 mM DTT; pH = 8.5). Bound proteins were eluted with elution buffer (buffer A supplemented with 300 mM imidazole). The eluate was diluted with 4 volumes of buffer A, centrifuged for 10 min at 20,000× *g* and 4 °C. Proteins were further purified by ion-exchange chromatography (IEX) using a Resource Q 1 mL column (Cytiva, Marlborough, MA, USA) connected to an ÄKTA purifier system (Cytiva, Marlborough, MA, USA). Bound proteins were eluted with a gradient of buffer B (25 mM Tris/HCl; 1000 mM NaCl; 5% (*v/v*) glycerol; 1 mM DTT; pH = 8.5). Fractions containing the protein of interest were concentrated using an Amicon 10 kDa concentrator (Merck Millipore, Burlington, MA, USA) and washed three times with size exclusion chromatography (SEC) buffer (20 mM Tris/Cl, 150 mM NaCl, pH = 7.9). For removal of the His-DHFR tag, the solution was incubated with 0.6 mg/mL TEV protease overnight at 4 °C. The TEV protease was N-terminally fused to a His tag to allow for subsequent removal using Ni-NTA columns. After the addition of 10 mM imidazole, Orf9b was obtained from the TEV digestion mix as an unbound pass-through fraction using a 1 mL HisTrap High Performance column (Sigma-Aldrich, St. Louis, MO, USA) connected to an ÄKTA Start system, while the His-tagged TEV protease and the His-DHFR-TEV part of the fusion protein remained bound to the column bed. After concentration of the Orf9b fraction using an Amicon 3 kDa concentrator (Merck Millipore, Burlington, MA, USA), the protein solution was subjected to size exclusion chromatography (SEC) using a Superdex 75 increase 10/300 GL column (Cytiva, Marlborough, MA, USA). A typical retention volume for Orf9b_WT_ and Orf9b_S53E_ during SEC was 11.6 mL, which corresponds to an approximate molecular mass of 28.7 kDa by comparison with a protein standard curve. Eluted fractions containing the target protein were concentrated using an Amicon 3 kDa concentrator (Merck Millipore, Burlington, MA, USA), and the protein concentration was determined by Bradford assay using a bovine serum albumin standard (2 mg/mL; Sigma-Aldrich, St. Louis, MO, USA) as a reference [49]. The solution containing purified Orf9b was divided into aliquots, frozen in liquid nitrogen, and stored at −70 °C until further use.

Human TOM70_111–608_ was purified as previously described, with minor modifications [24]. *E. coli* BL21 (DE3) cells were transformed with pProEx-HTA-TOM70_111–608_, and protein expression was induced at OD_600_ = 0.6 with 0.5 mM IPTG overnight at 16 °C. Cells were harvested and resuspended in lysis buffer (20 mM Tris/HCl; 500 mM NaCl; 10 mM imidazole; 2 mM PMSF; pH = 7.9) supplemented with 4.5 U/mL DNase I and protease inhibitors and incubated for 30 min at 4 °C. Cells were lysed by four passages through an Emulsiflex C5 homogenizer (Avestin, Ottawa, ON, Canada) and cleared from debris by centrifugation at 47,500× *g* for 30 min at 4 °C. After the addition of 10 mM imidazole, N-terminally His-tagged TOM70 was purified from the extract by affinity purification using Ni-NTA HisTrap High Performance columns (Sigma-Aldrich, St. Louis, MO, USA) and an ÄKTA Start protein purification system (Cytiva, Marlborough, MA, USA). Columns were washed with 10 cv wash buffer (20 mM Tris/HCl; 500 mM NaCl; 40 mM imidazole; pH = 7.9) and bound proteins were eluted with elution buffer (20 mM Tris/HCl; 500 mM NaCl; 300 mM imidazole; pH = 7.9). Protein-containing fractions were pooled and concentrated using an Amicon 30 kDa concentrator (Merck Millipore, Burlington, MA, USA) and washed three times with size exclusion chromatography (SEC) buffer (20 mM Tris/HCl; 150 mM NaCl; pH = 7.9). The protein was further purified using SEC with an ÄKTA purifier system equipped with a Superdex 200 increase 10/300 column (Cytiva, Marlborough, MA, USA). The protein concentration was determined by Bradford standard procedure using a bovine serum albumin standard (2 mg/mL; Sigma-Aldrich, St. Louis, MO, USA) as reference. The purified protein was divided into aliquots, frozen in liquid nitrogen, and stored at −70 °C until further use.

For protein purification in small batches for in vitro binding experiments, we used a standard protocol published by Brix et al. with minor modifications [23]. Briefly, after protein expression, *E. coli* cells were harvested by centrifugation, resuspended in 10 mM MOPS buffer (pH = 7.2) and frozen in small aliquots until further use. For protein purification, bacteria were resuspended in binding buffer (20 mM Tris/HCl; 500 mM NaCl; 10 mM imidazole; 2 mM PMSF; pH = 7.9), and cells were disrupted by three rounds of 10 pulses using a Sonifier S-250A cell disruptor (Branson Ultrasonics, Brookfield, CT, USA) equipped with a micro tip. Insoluble fragments were sedimented by centrifugation at 20,000× *g* for 30 min at 4 °C. Protino Ni-NTA agarose beads (Macherey-Nagel, Düren, Germany) were added to the supernatant and incubated on a rotation mixer at 4 °C for one hour. Afterward, Ni-NTA beads were washed five times with a standard wash buffer (20 mM Tris/Cl; 150 mM NaCl; 40 mM imidazole; 2 mM PMSF; pH = 7.9). The amounts of protein bound to Ni-NTA were determined by SDS-PAGE and comparison to a BSA standard (2 mg/mL; Sigma-Aldrich, St. Louis, MO, USA), Coomassie stain, and densiometric analysis. Isolated proteins were kept on ice until further use.

### 4.3. In Vitro Translation of Radiolabeled Proteins

Radiolabeled proteins were synthesized by coupled transcription and translation in commercially available reticulocyte lysate (TNT Coupled Reticulocyte Lysate System, L4600; Promega, Madison, WI, USA) in the presence of [^35^S]-methionine (added from a stock solution of 10 μCi/μL, SCM01/37; Hartmann Analytic, Braunschweig, Germany), following the instructions of the manufacturer. The reaction was carried out for 120 min at 30 °C. Reticulocyte lysate containing the radiolabeled precursor protein was clarified by centrifugation (1 h; 20,000× *g*; 4 °C) before use. Small proteins (<15 kDa) were precipitated with a saturated ammonium sulfate solution by incubation on ice for 20 min, followed by centrifugation for 20 min at 20,000× *g*, and resuspended in untreated reticulocyte lysate (L4151; Promega, Madison, WI, USA).

### 4.4. In Vitro Binding Assay

To analyze the binding of proteins to the isolated cytosolic domain of human TOM70 (amino acids 111–608), we used an in vitro binding assay modified according to Brix et al. [23]. The purified cytosolic domain of TOM70 bound to Ni-NTA agarose beads was washed three times with assay buffer (10 mM MOPS/KOH; 20 mM imidazole; 100 mM KCl; 1% BSA (*w/v*); pH = 7.2) prior to use. A reaction mix containing 50–250 pmol of purified TOM70 per 100 µL assay buffer was added to Mobicol columns (M1002; MoBiTec, Göttingen, Germany), and the reaction was started by addition of 7% (*v/v*) reticulocyte lysate containing the [^35^S]-methionine-labeled protein. After incubation for 40 min at 30 °C while shaking (750 rpm), the column bed was washed three times with assay buffer without BSA (10 mM MOPS/KOH; 20 mM imidazole; 100 mM KCl; pH = 7.2). Proteins were eluted by the addition of 300 µL elution buffer (20 mM Tris/HCl; 1 M imidazole; 500 mM NaCl; pH = 7.9), incubation for 3 min at 30 °C, and subsequent centrifugation at 250× *g* for one minute. Eluates were precipitated and washed, following a standard trichloroacetic acid (TCA) precipitation protocol, and analyzed by SDS-PAGE. Individual binding experiments were carried out with separate samples in individual columns, and internal standard samples (“Load”) were included in each experiment. Quantification of bound protein was performed using the software Aida Image Analyzer V.4.19 (Elysia-raytest GmbH, Straubenhardt, Germany). Unspecific binding was analyzed by incubation of radiolabeled proteins with Ni-NTA beads that had been incubated with *E. coli* lysate prior to the assay, and unspecific binding values were subtracted from quantified binding values. The typical amounts of radiolabeled proteins bound to the immobilized TOM70 variants in these assays varied between 0.5% and 5%.

To investigate the influence of purified Orf9b on the binding of target proteins, Ni-NTA-bound TOM70 was washed three times with assay buffer containing BSA (10 mM MOPS/KOH; 20 mM imidazole; 100 mM KCl; 1% BSA (*w/v*); pH = 7.2) and was subsequently incubated with a 10-fold molar amount of Orf9b_WT_ or Orf9b_S53E_. The beads were incubated at 30 °C while shaking (750 rpm) for 20 min and washed three times with assay buffer containing BSA (10 mM MOPS/KOH; 20 mM imidazole; 100 mM KCl; 1% BSA (*w/v*); pH = 7.2). Afterward, binding experiments using radiolabeled target proteins were performed as described above.

### 4.5. Trypsin Digestion

For trypsin digestion experiments, 5 µg/mL trypsin were added to either isolated proteins (5.5 µg/mL) or reticulocyte lysate containing radiolabeled proteins (20% (*v/v*) in a total volume of 100 µL). Samples were incubated in SEC buffer (20 mM Tris; 150 mM NaCl; pH = 7.9) and kept on ice during trypsin digestion. In the case of TOM70 pre-incubation, reticulocyte lysate containing radiolabeled proteins was incubated with isolated TOM70 at 25 °C for 10 min prior to the addition of the protease. The trypsin digestion was stopped by incubation at 95 °C for 5 min, and samples were subsequently prepared for SDS-PAGE.

### 4.6. Gel Electrophoresis

For SDS-polyacrylamide gel electrophoresis (SDS-PAGE), proteins were denatured in SDS-sample buffer (50 mM Tris/HCl; 1% (*w/v*) SDS; 5% (*v/v*) 2-mercaptoehtanol; 0.01% (*w/v*) bromophenol blue; 10% (*v/v*) glycerol) by incubation for 5 min at 95 °C. Samples were loaded on polyacrylamide gels (12.5% (*w/v*) acrylamide; 0.33% (*w/v*) bis-acrylamide; 350 mM BisTris; pH = 6.5) and electrophoresis was carried out at 100 V for 10 min and 150 V for 30–50 min in either MOPS buffer (50 mM MOPS; 50 mM Tris/HCl; 1 mM EDTA; 0.1% (*w/v*) SDS; 5 mM Na_2_SO_3_; pH = 7.0) or MES buffer (50 mM MES; 50 mM Tris/HCl; 1 mM EDTA; 0.1% (*w/v*) SDS; 5 mM Na_2_SO_3_; pH = 7.0). Gels were either dried and incubated on image plates for read out by a Bas1800 II phosphorimager (Fujifilm Europe GmbH, Düsseldorf, Germany) and analyzed by Aida Image Analyzer V 4.19 (Elysia-raytest GmbH, Straubenhardt, Germany) or stained with colloidal Coomassie (5% (*w/v*) aluminum sulfate 14–18 hydrate; 5% phosphoric acid; 10% (*v/v*) ethanol; 0.02% (*w/v*) Coomassie Brilliant Blue G-250) for 1 h to overnight and destained for 2 h with destaining solution (2% (*v/v*) phosphoric acid; 10% (*v/v*) ethanol).

Blue native gel electrophoresis was performed essentially as described by Schägger and von Jagow (1991) [50], with minor modifications using freshly prepared 6–16.5% linear gradient gels. Briefly, a 6% gel solution (6% (*w/v*) acrylamide mixture; 50 mM BisTris; 500 mM 6-aminohexanoic acid) and a 16.5% gel solution (16.5% (*w/v*) acrylamide mixture; 50 mM BisTris; 500 mM 6-aminohexanoic acid; 20% (*w/v*) glycerol) were prepared from a 49.5% (*w/v*) acrylamide mixture (48% (*w/v*) acrylamide; 1.5% (*w/v*) bis-acrylamide) and a 3x gel buffer (150 mM BisTris; 1.5 M 6-aminohexanoic acid; pH = 7.0). Polymerization was initiated by addition of 0.5% (*w/v*) APS (ammonium persulfate) from a freshly prepared 10% (*w/v*) APS stock solution and 0.1% (*v/v*) TEMED (tetramethyl-etylenediamine). Using equal volumes of 6% and 16.5% gel solutions, gels were cast using a gradient mixer under constant flow. Freshly prepared reticulocyte lysate samples containing [^35^S]-labeled proteins were centrifuged at 20,000× *g* for 1 h and 4 °C. Afterward, samples were supplemented with digitonin buffer (20 mM Tris/Cl; 0.1 mM EDTA; 50 mM NaCl; 10% glycerol (*w/v*); 1% digitonin (*w/v*); pH = 7.0) and 1/10 vol. sample buffer (100 mM BisTris; 500 mM 6-aminohexanoic acid; 5% (*w/v*) Coomassie Brilliant Blue G-250; pH = 7.0) and loaded onto gels. Electrophoresis was performed at 100 V for 1 h and 200 V with the current limited to 15 mA for 2–3 h at 4 °C, using anode buffer (50 mM BisTris; pH = 7.0) and cathode buffer (50 mM Tricin; 15 mM BisTris; 0.02% (*w/v*) Coomassie Brilliant Blue G-250; pH = 7.0). Gels were dried and incubated with image plates for read out by a Bas1800 II phosphorimager (Fujifilm Europe GmbH, Düsseldorf, Germany) and analyzed by Aida Image Analyzer V 4.19 (Elysia-raytest GmbH, Straubenhardt, Germany).

### 4.7. Western Blot

Samples were analyzed after SDS-PAGE using Western blot following a standard protocol. After gel electrophoresis, the proteins were transferred to a nitrocellulose membrane (Amersham Protran 0.2 µM NC; GE Healthcare, Chicago, IL, USA) at 300 mA for 1 h in blotting buffer (10 mM NaHCO_3_; 3 mM Na_2_CO_3_; 20% (*v/v*) methanol; 0.01% (*w/v*) SDS; pH = 7.4). Membranes were dried, washed twice with TBS (10 mM Tris/Cl; 150 mM NaCl; pH = 7.5), and blocked with 5% (*w/v*) non-fat dry milk powder in TBS overnight at 4 °C under constant agitation. After two wash steps with TBS-TT (20 mM Tris/Cl; 500 mM NaCl; 0.05% (*v/v*) Tween-20; 0.2% (*v/v*) Triton X-100; pH = 7.5) and one subsequent wash step with TBS, membranes were incubated with a monoclonal mouse anti-His antibody (Invitrogen MA1-21315, 1:1000 in TBS; Thermo Scientific, Waltham, MA, USA) for one hour at room temperature and constant agitation. Wash steps were repeated, and the membrane was incubated with a fluorophore-conjugated goat anti-mouse antibody (IRDye 800CW, 1:15,000 in 10% (*w/v*) non-fat dry milk powder in TBS; LI-COR Biosciences, Lincoln, NE, USA) for one hour at room temperature and constant agitation. Membranes were scanned after four wash steps with TBS-TT, and signals were detected using an Odyssey Infrared Imaging System (LI-COR Bioscience, Lincoln, NE, USA).

### 4.8. Cell Culture

Vero E6 cells were maintained and cultured as a monolayer in complete Dulbecco’s modified Eagles medium (DMEM) at 37 °C, 95% humidity, and in a 5% CO_2_ atmosphere. Once cells reached 90% confluency, cells were detached from culture dishes with a 0.05% trypsin solution for 10 min at 37 °C, resuspended in appropriate media (prewarmed), and either used experimentally or re-seeded for further expansion in new culture dishes. Cells were exclusively seeded onto collagen-coated dishes.

### 4.9. Transfection and Immunofluorescence Microscopy

For transfection, Vero E6 cells were seeded at a density of 120,000 cells per well onto sterile coverslips embedded in a 24-well plate, incubated for 4 h, and subsequently transfected with 0.8 µg of pCAGGS vector containing the constructs of interest using Lipofectamine 2000 (100 µL Opti-MEM reduced serum media, 0.8 µg vector, 2.4 µg Lipofectamin 2000 per well). At 24 h post-transfection, cells were fixated (3% PFA), permeabilized (0.2% TritonX-100), blocked (5% horse serum), and stained with an anti-COXIV antibody (1:400; Cat. No. 4850S; Cell Signaling Technologies, Danvers, MA, USA). Cell nuclei were stained with DAPI. Before imaging, coverslips were mounted onto cover slides with 6 µL of Fluoromount. Images were acquired with a standard wide-field fluorescence microscope (BZ-X800E; Keyence, Osaka, Japan) in combination with a 60 × 1.40 numerical aperture (NA) oil-objective. The microscope is equipped with an 80 W metal halide lamp for the excitation of the DNA dye and a 2/3” monochrome CCD element (4080 × 3060 pixels per acquired image). DAPI (358 nm), green (488 nm), and red (568 nm) signals were acquired sequentially using BZ-X Filter DAPI, BZ-X Filter GFP, and BZ-X Filter TexasRed, respectively. Z-stack images were acquired with a 0.5 µm pitch, and full focus images were generated using BZ-X800 Analyzer.Ink (Keyence, Osaka, Japan) software.

### 4.10. Image Analysis

Gray value profiles in Figure 2 were analyzed using ImageJ software (Fiji) [51] and normalized to the smallest and largest values in each data set with GraphPad Prism version 9.1.2 for Windows (GraphPad Software, San Diego, CA, USA). Figures were generated using Illustrator CC 2021 (Adobe Inc., San Jose, CA, USA).

### 4.11. Statistical Analysis

For statistical analysis, the normality distribution of values was first tested by a Shapiro–Wilk test except for Figure 4F,G, where a Kolmogorov–Smirnov test was used. Normality distribution was confirmed for all tests. According to this result, two-tailed *t*-tests were performed for the comparison of two groups. Alternatively, one-way ANOVA was performed for statistical analysis of three or more groups. In Figure 1K and Figure 3C, one-way ANOVA was performed by Dunnett’s multiple comparisons test, with control group as indicated. In Figure 4E, one-way ANOVA was performed by Šídák’s multiple comparisons test to compare data sets in the two groups. All bar graphs contain error bars that display the standard error of means (SEMs). Statistical analysis was performed using GraphPad Prism 9 version 9.1.2 (GraphPad Software, San Diego, CA, USA).

## Figures and Tables

**Table 1:** Plasmids used in this study.

Plasmid	Source
pProEx-HTA-TOM70(111–608)	Young et al., 2003; [31]
pProEx-HTA-TOM70(111–608)_R192A_	This study
pProEx-HTA-TOM70(111–608)_E477A_	This study
pCDNA3-Hsp90-HA	García-Cardeña et al., 1998; [47]
pYES2-Hsp90	This study
pDONR334-SARS-CoV2 Orf9b	Kim et al., 2020; [48]
pProEx-His-DHFR-TEV-Orf9b	This study
pProEx-His-DHFR-TEV-Orf9b_S53E_	This study
pYES2-Orf9b	This study
pYES2-Orf9b_S53E_	This study
pYES2-Orf9b_S53A_	This study
pYES2-Orf9b_1–76_	This study
pYES2-Orf9b_33–97_	This study
pYES2-Orf9b-DHFR	This study
pYES2-Orf9b_S53E_-DHFR	This study
pYES2-Orf9b_S53A_-DHFR	This study
pYES2-AAC3	This study
pYES2-DHFR	This study
pCAGGS-Orf9b-FLAG-EGFP	This study
pCAGGS-Orf9b_S53E_-FLAG-EGFP	This study

## Data Availability

The data presented in this study are available upon reasonable request from the corresponding author.

## Supplementary Materials

The following are available online at https://www.mdpi.com/article/10.3390/ijms22179233/s1.

## Abbreviations

[^35^S]	Radiolabeling by ^35^S-methionine residues
AAC3	Human ADP/ATP carrier 3
BN-PAGE	Blue native polyacrylamide gel electrophoresis
Co-IP	Co-immunoprecipitation
COVID19	Coronavirus disease 2019
COXIV	Subunit of the cytochrome c oxidase
Cryo-EM	Cryogenic electron microscopy
DAPI	4′,6-diamidino-2-phenylindole
DHFR	Mouse dihydrofolate reductase
EGFP	Enhanced green fluorescent protein
Hsp90	Human heat shock protein of 90 kDa
IEX	Ion exchange chromatography
IFN-I	Interferon type I
IMAC	Immobilized metal affinity chromatography
IRF3	Interferon regulatory factor 3
ITC	Isothermal titration calorimetry
kDa	Kilo Dalton
MAVS	Mitochondrial antiviral-signaling protein
Ni-NTA	Nickel-nitrilotriacetic acid
Orf9b	Protein encoded by the open reading frame 9b of SARS-CoV-2
PBMC	Peripheral blood mononuclear cells
RIG-I	Retinoic acid-inducible gene I
RL	Rabbit reticulocyte lysate
SARS-CoV-2	Severe acute respiratory syndrome coronavirus 2
SDS-PAGE	Sodium dodecyl sulfate-polyacrylamide gel electrophoresis
SEC	Size exclusion chromatography
SEM	Standard error of the means
TBK1	TANK-binding kinase 1
TEV	*Tobacco etch virus* nuclear-inclusion-a endopeptidase
TOM	Translocase of the outer mitochondrial membrane
TOM70	Human TOM protein of 70 kDa
Tom70	Yeast TOM Protein of 70 kDa
Tom71	Yeast paralogue of Tom70
TPR	Tetratricopeptide repeat
