# Supplementary material for: Phosphorylation of SARS-CoV-2 Orf9b Regulates Its Targeting to Two Binding Sites in TOM70 and Recruitment of Hsp90
Source: Int J Mol Sci. 2021 Aug 26;22(17):9233. doi: 10.3390/ijms22179233 (PMC8430508; doi:10.3390/ijms22179233)
Supplement: Supplementary file 1 [file ijms-22-09233-s001.zip › ijms-1305704-supplementary.pdf]

Supplementary Material
Brandherm et al.

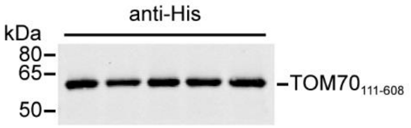

Figure S1: Labeling of TOM70$_{111-608}$ after elution from Ni-NTA beads. In five parallel samples, Ni-NTA beads containing 250 pmol TOM70$_{111-608}$ were washed three times with assay buffer containing 1 % (w/v) BSA and subsequently incubated with assay buffer for 40 min at 30 °C under constant shaking (750 rpm). The samples were cooled on ice, washed, and bound TOM70$_{111-608}$ was eluted and precipitated as described in Materials and Methods (Binding Assay). From each sample, 20 % were analyzed by SDS-PAGE, western blotting and immunolabelling, using an antibody directed against the N-terminal His tag of TOM70$_{111-608}$.

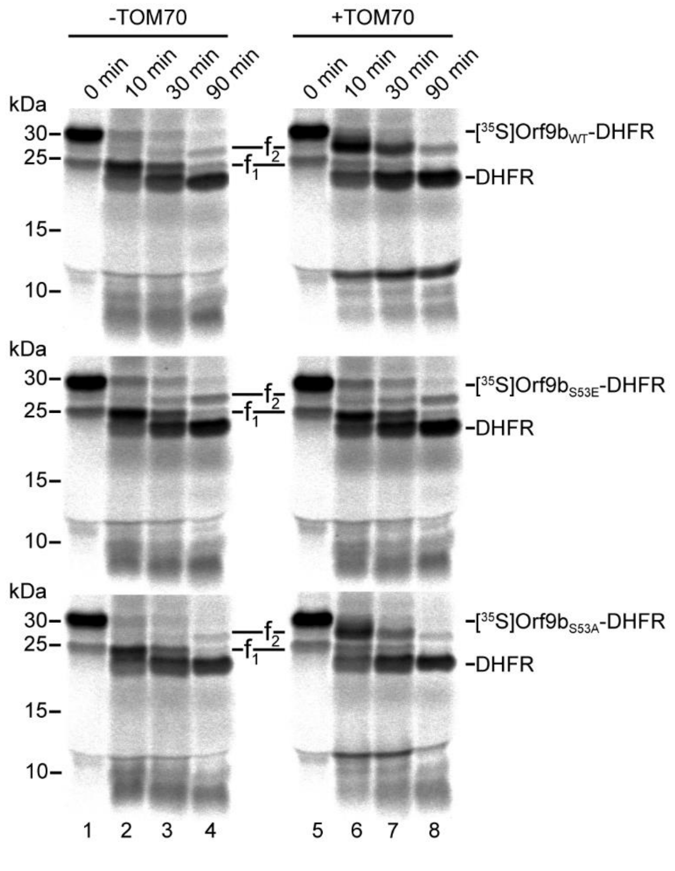

Figure S2: Degradation by trypsin of [^{35}S]Orf9b-DHFR fusion proteins in the absence or presence of isolated TOM70$_{111-608}$. The protein [^{35}S]Orf9b$_{WT}$-DHFR, [^{35}S]Orf9b$_{S53E}$-DHFR and [^{35}S]Orf9b$_{S53A}$-DHFR were synthesized in reticulocyte lysate and subsequently incubated with purified TOM70 (final concentration 0.1 mg/ml) for 10 min at 25 °C. Following addition of trypsin (5 µg/ml), the samples were incubated for 0, 10, 30, and 90 min on ice. Proteolysis was stopped by incubation for 5 min at 95 °C and degradation products were analyzed by SDS-PAGE. Radiolabeled proteins were visualized using a Bas1800 II phosphorimager (Fujifilm Europe GmbH, Düsseldorf, Germany) and the program Aida Image Analyzer V 4.19. Prominent degradation products are indicated as f1 (fragment 1) or f2 (fragment 2).

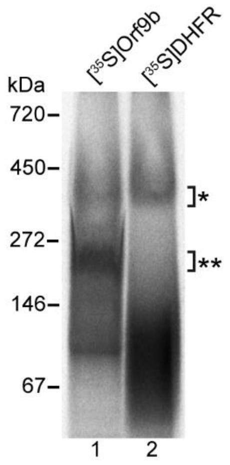

Figure S3: Analysis of reticulocyte lysates containing radiolabeled [^{35}S]Orf9b and [^{35}S]DHFR by blue native polyacrylamide gel electrophoresis (BN-PAGE). After translation of [^{35}S]Orf9b and [^{35}S]DHFR in reticulocyte lysate and removal of aggregates by centrifugation (1 h, 20,000 x *g*, 4 °C), 3 µl of each lysate were diluted and adjusted to a final volume of 40 µl. [^{35}S]Orf9b and [^{35}S]DHFR samples were subjected to analysis by BN-PAGE. The gel was dried and radiolabeled proteins were detected autoradiographically. Asterisks: * non-specific protein complex of the reticulocyte lysate; ** protein complex of Orf9b [21, 22].
